# High levels of multiresistance in quinolone resistant urinary tract isolates of *Escherichia coli* from Norway; a non clonal phenomen?

**DOI:** 10.1186/1756-0500-7-376

**Published:** 2014-06-19

**Authors:** Linda Strand, Andrew Jenkins, Ingrid Høgli Henriksen, Anne Gry Allum, Nils Grude, Bjørn Erik Kristiansen

**Affiliations:** 1 Unilabs Telelab, Skien, Norway; 2 Department of Environmental and Health Sciences, Telemark University College, Bø, Norway; 3 Department of Microbiology and Virology, University of Tromsø, Tromsø, Norway; 4 Present address: Telemark Hospital, Medical and Environmental Genetics Unit, Skien, Norway; 5 Present address: Department of Medical Microbiology, Vestfold Trust Hospital, Tønsberg, Norway

**Keywords:** Multiresistance, *E. coli*, Clone, Phylogenetic group, CGA, Ciprofloxacin, Nalidixic acid, PFGE

## Abstract

**Background:**

The problem of emerging ciprofloxacin resistance is compounded by its frequent association with multiresistance, the reason for which is not fully understood. In this study we compare multiresistance, clonal similarities and phylogenetic group in urinary tract isolates of *Escherichia coli* sensitive and resistant to the quinolone antimicrobials nalidixic acid and ciprofloxacin.

**Results:**

Quinolone resistant isolates were more resistant to non-quinolone antibiotics than sensitive isolates, with resistance to ampicillin, mecillinam, sulphonamide, trimethoprim, tetracycline, kanamycin and chloramphenicol significantly increased. Fifty-one percent of quinolone-resistant isolates were multiresistant. Although multiresistance was most prevalent (63%) in isolates showing high-level ciprofloxacin resistance, it was still highly prevalent (41%) in nalidixic acid resistant isolates with low-level ciprofloxacin resistance. Multiresistance was more frequent among singleton isolates (61%) than clonal isolates (40%) of quinolone resistant *Escherichia coli.* Ciprofloxacin resistance was associated with certain specific clones, among them the globally distributed clonal Group A. However, there was no significant difference in the overall degree of clonality between quinolone sensitive and resistant isolates. Ciprofloxacin resistance was positively associated with phylogroup D and negatively associated with phylogroup B2. This correlation was not associated with clonal isolates.

**Conclusion:**

This study supports earlier findings of association between ciprofloxacin resistance and resistance to other antibiotics. The prevalence of multiresistance in quinolone-resistant isolates that have not yet developed high-level ciprofloxacin resistance suggest that multiresistance arises early in the development of quinolone resistance. This is consistent with exposure to quinolones causing quinolone resistance by mutations and mobilization of multiresistance elements by induction of the SOS response. The spread of clones seems to be less important than previously reported in regard to emergence of quinolone resistance and multiresistance as both are associated primarily with singleton isolates.

## Background

Since the broad spectrum fluoroquinolone antibiotics were introduced in clinical practice, fluoroquinolone resistant *E. coli* (FQREC) strains have been isolated with increasing frequency, with prevalence as high as 25 – 50% being reported from some southern European countries. In Norway the prevalence of quinolone resistance, although still low, has doubled to 9.7% in the period 2005 – 2009 [1].

Quinolone resistance is principally due to mutations in the DNA gyrase gene *gyrA*. Single mutations result in nalidixic acid resistance and moderately raised ciprofloxacin resistance, while full resistance to ciprofloxacin requires two *gyrA* mutations. Mutations in *parC*[2-4], mutations that effect efflux pumps or cell permeability and plasmid-borne factors may also contribute to resistance [5-7].

The problem of emerging ciprofloxacin resistance is further compounded by its frequent association with multiresistance to other antibiotics [8-13]. The reason for this association is not understood, but several possibilities may be considered. Ciprofloxacin-resistant isolates have been reported to show a high level of clonality [8,14] and resistance has emerged in the multiresistant uropathogenic clonal group A (CGA) [15-20], which suggests a contribution from the spread of multiresistant clones under selection pressure. The agricultural sector has also been suggested as a source of ciprofloxacin resistance [21], in which case multiresistance might arise at source, driven by the extensive use of antibiotic additives as growth stimulants in animal feed. An association between multiresistance and ciprofloxacin resistance might be explained if ciprofloxacin tends to be chosen when therapy with first-line preparations fails due to multiresistance in the target organism. Lastly, quinolones are mutagenic and induce the SOS response which in turn activates the mobility of transposable elements [22-24], suggesting that chromosomal mutations and/or the spread of mobile resistance factors might be involved.

In this study we investigate multiresistance and clonal similarities in ciprofloxacin-sensitive and ciprofloxacin-resistant urinary tract isolates of *E. coli*, as well as the nalidixic-acid resistant isolates that are thought to be their precursors. Our results suggest that multiresistance emerges early in the development of ciprofloxacin resistance and place constraints on several of the possible explanations for association between ciprofloxacin resistance and multiresistance. We also reinvestigate our earlier findings suggesting fluoroquinolone resistance and clonal spread and the emergence of fluoroquinolone resistance in clonal group A [20].

## Results

### Ciprofloxacin MIC distribution

**Figure 1:**
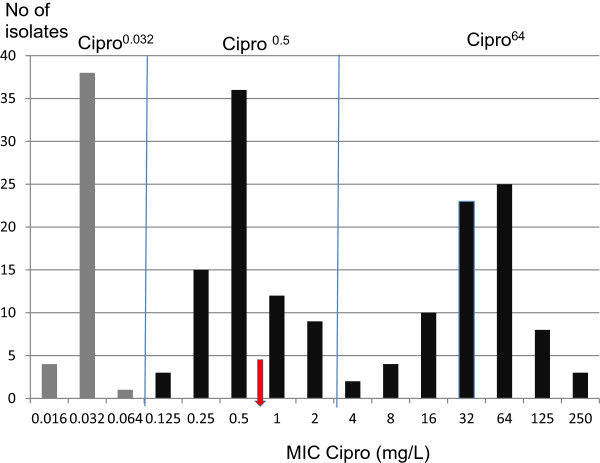
**Trimodal distribution of ciprofloxacin MIC.** Nalidixic acid sensitive isolates are shown in grey. Vertical lines delineate the subdivisions used for analysis in the rest of the article. The arrow indicates the EUCAST breakpoint between clinical resistance and sensitivity [1].

Figure 1 shows the ciprofloxacin MIC distribution of the isolates. The isolates are clearly separated into three subpopulations with peak MICs of 0.032, 0.5 and 64 mg/L. The first population consists of the nalidixic acid sensitive isolates, while the second two contain the nalidixic acid resistant isolates. We have chosen to divide our material on the basis of the natural divisions between the three subpopulations at 0.064 - 0.125 and 2–4 mg/L, rather than the clinical breakpoints, which do not coincide with the natural divisions in this material. In order to avoid confusion with the established sensitive-intermediate-resistant terminology, we refer to our three subpopulations as Cipro^0.032^- nalidixic acid sensitive isolates with ciprofloxacin MIC ≤ 0.064 and modal MIC 0.032 mg/L (N = 43); Cipro^0.5^ – nalidixic acid resistant isolates with ciprofloxacin MIC ≥ 0.125 and ≤ 2 and modal MIC 0.5 mg/L (N = 75) and Cipro^64^ – nalidixic acid resistant, ciprofloxacin resistant isolates with ciprofloxacin MIC ≥ 4 and modal MIC 32–64 mg/L (N = 75). Cipro^0.5^ and Cipro^64^ isolates are collectively referred to as nalidixic acid resistant isolates.

### Resistance to non-quinolone antibiotics

Nalidixic acid resistant isolates (Cipro^0.5^ and Cipro^64^) expressed a greater number of non-quinolone antibiotic resistances (mean = 3.5) than nalidixic acid sensitive (Cipro^0.032^) isolates (mean = 0.4; p < 0.001, *t*-test), with resistance to all antibiotics tested except nitrofurantoin significantly increased (z-test, p < 0.05). There was also a slight, but significant difference in the number of resistances between Cipro^0.5^ (mean = 3.2) and Cipro^64^ isolates (mean = 3.8; p < 0.05), with significant increase in resistance to trimethoprim and sulphonamide (z-test, p < 0.05). Antibiotic resistances of the isolates are shown in Table 1.

**Table 1:** Antibiotic resistances

	**Cipro**^**0.032 **^**(n = 43)**			**Cipro**^**0.5 **^**(n = 75)**			**Cipro**^**64 **^**(n = 75)**		
Antibiotic	S	I	R	S	I	R	S	I	R
Ampicillin	-	37	6	-	22	53	-	16	59
Mecillinam	41	-	2	62	-	13	60	-	15
Kanamycin	42	-	1	66	-	9	61	-	14
Chloramphenicol	41	-	2	50	-	25	45	-	30
Tetracycline	38	-	5	27	1	47	19	3	53
Nitrofurantoin	43	-	-	74	-	1	74	-	1
Sulphonamide	34	3	6	22	1	52	13	0	62
Trimethoprim	38	-	5	32	1	42	20	-	55

Cutoff values between sensitive, intermediate and resistant were: Ampicillin: I >0.5, R >8; Mecillinam: R >8; Chloramphenicol: R >8; Nitrofurantoin: R >64; Trimethoprim: I >2, R >4 [25]. Tetracycline: I >4, R >8; Sulphonamide: I >64, R >128 [26]. Kanamycin: R >10.

Fifty-one percent (77/150) of the nalidixic acid resistant isolates were multiresistant, as defined by resistance to four classes of antibiotics in addition to quinolones; 17% (25/150) of the isolates were resistant to five, 6% (9/150) to six and one to seven other antibiotic classes; this isolate showed intermediate susceptibility to mecillinam and was otherwise fully resistant to all antibiotics tested including ciprofloxacin. Eight isolates were fully susceptible only to nitrofurantoin.

Forty percent (30/75) of Cipro^0.5^ were multiresistant and 63% (47/75) of Cipro^64^ were multiresistant as defined by resistance to four classes of antibiotics in addition to quinolones. Fourty-nine percent of isolates conforming to the standard definition of ciprofloxacin resistance (MIC ≥ 2 mg/L) were multiresistant.

18% (27/150) of the nalidixic acid resistant isolates shared a distinctive ampicillin-chloramphenicol-sulphonamide-tetracycline-trimethoprim multidrug resistance phenotype; these were approximately equally distributed between Cipro^0.5^ (56%) and Cipro^64^ (44%).

Only one nalidixic acid sensitive isolate was multiresistant.

### Clonal distribution and phylogroup

**Figure 2:**
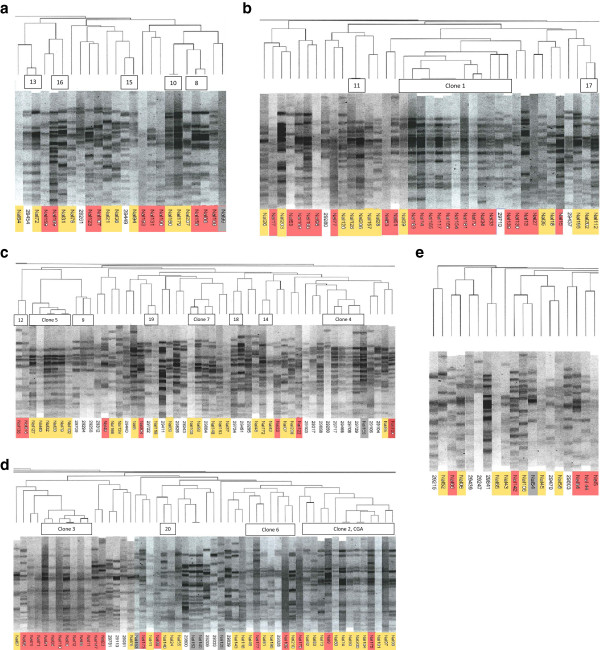
**XbaI PFGE patterns of the isolates. (a-e)** Highlighting of the isolate code indicates cipro^64^ (red), cipro^0.5^ (yellow) and cipro^0.032^ (not highlighted). Grey indicates that the sample is excluded from the study. Panels a - e are parts of a single image which has been divided for convenience of presentation. The left border of panel a is the right border of panel b, and so forth. Clone numbers are placed between the dendrogram and the gel pictures. In a few cases, patterns grouped with a clone in the dendrogram do not satisfy our criteria for clone inclusion (<7 bands difference from the prototype). Therefore the number of patterns marked may differ from the number given in Table 2.

Pulsed-field gel electrophoresis (PFGE) analysis identified 20 clones containing two to 12 isolates (Table 2, Figure 2). Seventy-eight (43%) of the typable isolates were clonal and 105 (57%) were singletons. Ten isolates were non-typable due to DNA degradation; these were excluded from statistical analyses of clonal groups. All ten non-typable isolates were of phylogenetic group D, resistant to nalidixic acid, ciprofloxacin (MIC 32–64 mg/L) and sulphonamide and lactose non-fermenting. Eight of these isolates were also resistant to trimethoprim, and seven were resistant to tetracycline and ampicillin. Five (50%) showed resistance against four or more antibiotics in addition to quinolones.

**Table 2:** Ciprofloxacin resistance and phylogenetic groups of clones

	**N**	**Cipro**^**0.032**^	**Cipro**^**0.5**^	**Cipro**^**64**^	**Phylogroup**	
Clone 1	12	-	1	11	B2	*Includes outbreak*
Clone 2	12	-	10	2	D	*CGA*
Clone 3	7	-	-	7	D	
Clone 4	5	5	-	-	B2	
Clone 5	6	-	6	-	B2	
Clone 6	4	1	3	-	D	
Clone 7	4	1	3	-	B2	
Clone 8	3	-	1	2	A	
Clone 9	3	3	-	-	B1	
Clone 10	2	-	2	-	B2	
Clone 11	2	-	2	-	B2	
Clone 12	2	-	-	2	B1	
Clone 13	2	1	1	-	A	
Clone 14	2	-	2	-	B2	
Clone 15	2	1	1	-	B2	
Clone 16	2	-	1	1	A	
Clone 17	2	-	2	-	A	
Clone 18	2	2	-	-	D	
Clone 19	2	1	1	-	B2	
Clone 20	2	-	2	-	D	

The two largest clones, clones 1 and 2, contained 12 isolates each and represented 13% (24/183) of the material. All clone 1 and 2 isolates were nalidixic acid resistant. Clone 1 included four isolates from a suspected outbreak at an old people’s home. Clone 2 was identified as clonal group A. All clones were homogeneous with respect to phylogenetic group.

**Figure 3:**
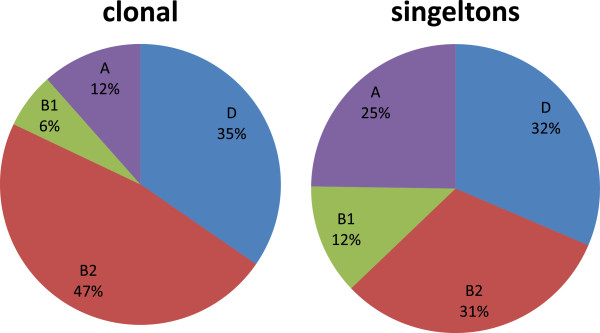
Distribution of phylogenetic groups A, B1, B2 and D among clonal and singleton isolates.

There was a significant association between clonality and phylogenetic group (Chi-square = 8.850 with 3 degrees of freedom; p = 0.040) (Figure 3). This can be attributed to the greater prevalence of phylogroup B2 among clonal isolates (+16% (CI_95_: 2-30%) p = 0.03, z-test) and of phylogroup A among singleton isolates (+13% (CI_95_:2-25%) p = 0.04, z-test). The proportion of phylogroup B1 and D was similar among clonal and singleton isolates (Figure 3) and there was no significant difference in clonality between these groups.

**Figure 4:**
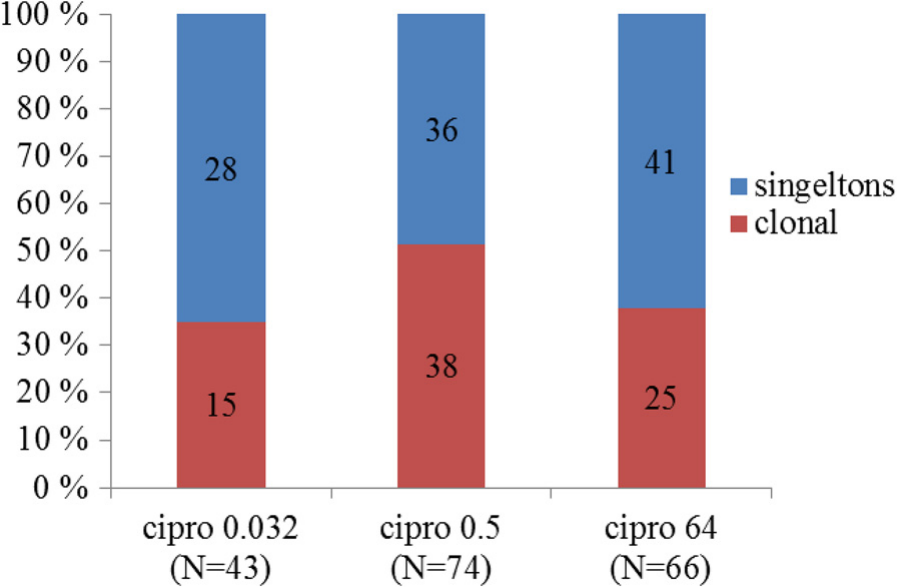
**Distribution of clonal and singleton isolates among cipro**^**0.032**^**, cipro**^**0.5 **^**and cipro**^**64 **^**isolates.**

There were no significant differences in the degree of clonality between Cipro^0.032^, Cipro^0.5^ and Cipro^64^ isolates (Chi-square (2 d.f.) = 3.966, p = 0.138), or between nalidixic acid sensitive and resistant isolates (Chi-square = 0.994; P = 0.319), see Figure 4. Ciprofloxacin resistance was, however, associated with specific clones. Two clones contained only Cipro^64^ isolates; six contained only Cipro^0.5^ isolates and three contained only Cipro^0.032^ isolates; five groups contained Cipro^0.032^ and Cipro^0.5^ isolates and four contained Cipro^0.5^ and Cipro^64^ isolates; no clone contained both Cipro^0.032^ and Cipro^64^ isolates (Table 2).

### Clonal group A (CGA)

All twelve CGA isolates were resistant to nalidixic acid with ciprofloxacin MICs from 0.25 to 8 mg/L, broadly coincident with the Cipro^0.5^ distribution. Four were ciprofloxacin resistant (MIC ≥ 2 mg/L). Eight isolates were multiresistant: four were resistant to four, three to five and one to six other antibiotics. This last isolate was fully sensitive only to nitrofurantoin.

Three isolates shared the ampicillin-chloramphenicol-sulphonamide-tetracycline-trimethoprim multidrug resistance phenotype predominant in other multiresistant nalidixic acid resistant isolates.

### Quinolone resistance and phylogroup

**Figure 5:**
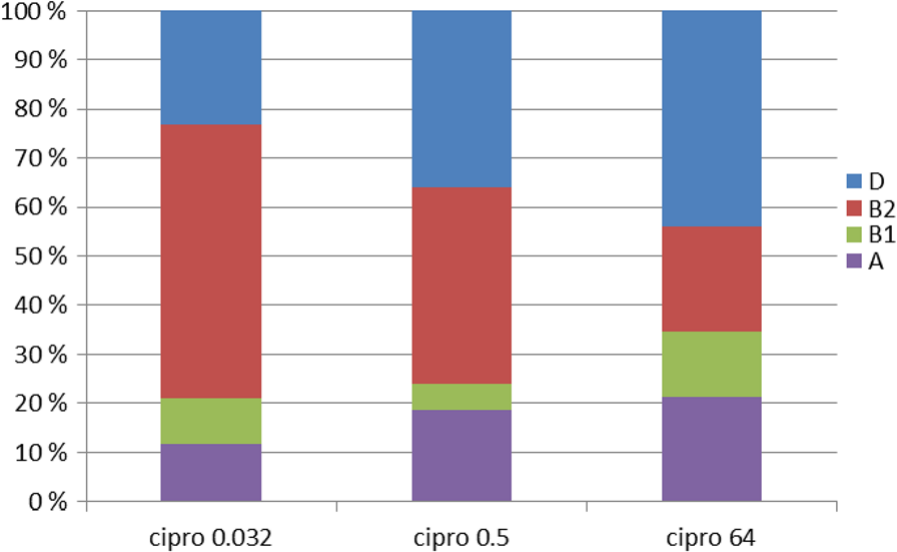
**Distribution of phylogenetic groups among cipro**^**0.032**^**, cipro**^**0.5 **^**and cipro**^**64 **^**isolates.**

Ciprofloxacin resistance was positively associated with phylogroup D and negatively associated with phylogroup B2 (Figure 5). Phylogroup D was significantly more prevalent (44%, 33/75) among Cipro^64^ isolates than among Cipro^0.032^ isolates (23%, 10/43) (+21%, CI_95_: 2%-38%; z-test, p = 0.04) while the prevalence of phylogroup B2 was lower (21%, 16/75) in Cipro^64^ than in Cipro^0.032^ (56%, 24/43) (−35%, CI_95_: 17%-53%; z-test, p < 0.001).

In order to investigate whether this is due to the effect of clones, we compared the correlation between phylogroup and quinolone resistance in singleton and clonal isolates. Phylogroup was correlated with quinolone resistance in singleton isolates (chi-squared, 6 d.f. = 17.009, p = 0.009), but not in clonal isolates (Chi-square, 6 d.f. = 8.626; p = 0.196). Phylogroup B2 was 45% (CI_95_: 23%-67%) less prevalent in Cipro^64^ than in Cipro^0.032^ singleton isolates (p < 0.001, z-test); the prevalence of phylogroups A, B1 and D was increased but did not achieve statistical significance (p > 0.2, z-test).

### Multiresistance in clonal and singleton isolates

In quinolone-resistant isolates, multiresistance, as defined by resistance to four classes of non-quinolone antibiotics, was found in 25 of 63 (40%) clonal isolates and 47/77 (61%) singleton isolates. This is statistically significant (p = 0.019). Non-typable isolates were not included.

## Discussion

This study supports earlier findings [8-13] of an association between ciprofloxacin resistance and resistance to other antibiotics, frequently in the form of multiresistance. Association was found with resistance to all antibiotics tested except nitrofurantoin. This, however, applies not only to ciprofloxacin-resistant isolates but also to nalidixic acid resistant isolates with low ciprofloxacin resistance levels, which may be presumed to be isolates with only one *gyrA* mutation. This indicates that multiresistance arises at an early stage in the stepwise development of ciprofloxacin resistance. As far as we are aware, association between nalidixic acid resistance and multiresistance has not previously been reported.

Eight quinolone-resistant isolates were susceptible only to nitrofurantoin; one isolate showed only intermediate susceptibility to mecillinam and was otherwise fully resistant to all antibiotics tested. These isolates were not clonally related. However, one isolate belonged to clonal group A and resembled two highly resistant CGA isolates previously isolated by us in 2003 [20].

The cause of the association between multiresistance and quinolone resistance is not known. However, a model based on spread of multiresistant clones is not consistent with our data. If the emergence of multiresistant quinolone-resistant isolates were due to clonal spread, we would expect multiresistance to be more prevalent among clonal isolates. This is the reverse of what we observe in this study. Singleton isolates are significantly more multiresistant than clonal isolates.

Neither do our results confirm previous findings of increased clonality among ciprofloxacin-resistant isolates [8,14]. Ciprofloxacin resistant isolates were no more clonal than sensitive isolates. Ciprofloxacin resistant clones did however occur, suggesting that clonal spread does make some contribution to the spread of ciprofloxacin resistance. Cagnacci et al. assigned 34% of 148 ciprofloxacin resistant isolates to two clonal groups using a combination of methods including multilocus sequence typing (MLST) [14]. This is a higher degree of clonality than we observe, despite their collection being both more geographically dispersed and collected over a longer time period. However their study includes material from the Mediterranean region where quinolone resistance is well established, while our material is from a low-prevalence region where emergence of quinolone resistance may still be dominant over spread.

Clonal group A was the second most prevalent clone found in our material, representing 6% (12/193) of the isolates. All CGA isolates were quinolone resistant and one third were ciprofloxacin resistant, confirming the emergence of fluoroquinolone resistance in CGA that we [20] and others [11] have previously observed. Although CGA is considered a multiresistant clone, CGA isolates in this material were not significantly more multiresistant than other quinolone resistant isolates (p > 0.5, z-test). In 2001, CGA comprised 19% of quinolone sensitive isolates, while in this material, from 2005, quinolone-sensitive CGA was not found. This difference is statistically significant (p = 0.011, z-test) and strongly suggests that a shift to quinolone resistance in CGA has occurred.

The four largest clones among the quinolone resistant isolates, clones 1, 2 (CGA, discussed above), 3 and 5 belonged to the uropathogenic phylogenetic groups B2 and D and comprised 25% of the quinolone-resistant isolates. The twelve clone 1 isolates belonged to phylogenetic group B2. Four of these isolates were epidemiologically associated. Clone 3 (7 isolates) belonged to phylogroup D. Clone 5 (6 isolates) belonged to phylogenetic group B2. Clones 1, 2 (CGA) and 3 were also present among fluoroquinolone resistant isolates from 2003 [8]. The clones did not display uniform resistance patterns, although all of clone 3 and 66% of clone 2 (CGA) isolates were trimethoprim-sulfonamide resistant.

Quinolone resistance was positively correlated with phylogenetic group D and negatively correlated with phylogenetic group B2 as has previously been reported [27-29]. This correlation was confined to non-clonal isolates, which implies that phylogroup D more readily acquires quinolone resistance mutations than phylogroup B2 and that the spread of clones is of lesser importance, in contrast to what has previously been suggested [8,14].

The predominant multiresistance phenotype, resistance to ampicillin, sulphonamide, trimethoprim, tetracycline and chloramphenicol was found in 19% of quinolone resistant isolates. It was widely disseminated among clonally unrelated isolates. This resembles the resistance pattern described in outbreak strains of O15:K52:H1 and CGA [16,17] and was found in three of the CGA isolates in this study. The genes giving this resistance phenotype have been located in a chromosomal integration hot spot in CGA by Lescat and coworkers [30].

This multiresistance pattern is one typically associated with transposable elements and plasmids, suggesting an association between quinolone resistance and mobile genetic elements and lending support to the idea that multiresistance arises by the mobilization of transposable elements caused by induction of the SOS response during quinolone therapy, an effect that has been suggested by several authors [22-24]. In this model, it is exposure to quinolones that causes mobilization of multiresistance elements, which is consistent with an early emergence of multiresistance, as observed in this study.

Fluoroquinolones are popular antibiotics; easily administered, cheap, broadly applicable, quick acting and showing low toxicity. However, in view of the emergence of resistance in pathogenic clonal groups such as CGA and the tendency of fluoroquinolones to provoke multiresistance, some caution in their use and monitoring of their effects is indicated.

## Conclusions

This study supports earlier findings of association between ciprofloxacin resistance and resistance to other antibiotics. We found that emergence of multiresistance arises early in the development of quinolone resistance and is mostly associated with singleton isolates. This is consistent with exposure to quinolones causing quinolone resistance by mutations and mobilization of multiresistance elements by induction of the SOS response. The spread of clones seems to be less important than previously reported in regard to emergence of quinolone resistance and multiresistance.

## Methods

### *E. coli* isolates

*E. coli* isolates were collected from community urine samples submitted to Telelab, a microbiology laboratory serving hospitals and primary practices in the counties of Telemark and Vestfold, south-eastern Norway for routine bacteriological analysis and showing significant bacteruria (pure culture of ≥ 10 000 bacteria/ml). The material consisted of 150 consecutive nalidixic acid resistant isolates and 43 randomly-chosen nalidixic acid sensitive isolates collected in the same period in 2005. *E. coli* was identified by colony morphology and biochemical profile using the three tube test [31].

### Clonal analysis

Pulsed-field gel electrophoresis using restriction endonuclease XbaI was performed as previously described [8,32]. Gels were photographed, scanned as TIFF files, and imported into Gelcompar software (Applied Maths, Sint-Martin-Latem, Belgium). A dendrogram was constructed using band-based comparisons with Dice coefficient and cluster analysis with the unweighted pair-group method with arithmetic average (UPGMA); 1% position tolerance and 3% optimization settings were used. PFGE patterns differing by less than seven bands from prototype members were assigned to the same clone.

### Phylogrouping

Phylogrouping by triplex-PCR was performed as previously described [8,32].

### Resistance

MICs of ampicillin, mecillinam, nitrofurantoin, sulphonamide, trimethoprim, tetracycline, nalidixic acid, ciprofloxacin, kanamycin and chloramphenicol were measured by agar dilution on iso-sensitest medium (Oxoid, Basingstoke, UK). MIC was defined as the lowest concentration that completely inhibited growth.

### Clonal group A

Isolates were identified by detection of the *fumC* SNP C288T by PCR as previously described [18,20].

### Statistical analyses

The Pearson Chi-square test was used to detect significant differences in clonality and phylogenetic distribution between nalidixic acid resistant and sensitive isolates. The *t*-test was used to test for significant difference in multiplicity of antibiotic resistances. The z-test was used to compare proportions.

## Competing interests

The authors report no competing interest.

## Authors’ contributions

Phylogrouping was done by IHH and AGA. PFGE was done by IHH. CGA testing was done by AGA and IHH. Antibiotic resistance testing was conducted by IHH. The study was planned by BEK, NG, AJ and LS. Data analysis, statistical analysis and drafting of the manuscript were done by LS with the assistance of AJ. NG and BEK provided critical comments on the manuscript. All authors read and approved the final manuscript.
